# Knowledge driven decomposition of tumor expression profiles

**DOI:** 10.1186/1471-2105-10-S1-S20

**Published:** 2009-01-30

**Authors:** Martin H van Vliet, Lodewyk FA Wessels, Marcel JT Reinders

**Affiliations:** 1 Information and Communication Theory Group, Faculty of Electrical Engineering, Mathematics and Computer Science, Delft University of Technology, Mekelweg 4, 2628 CD Delft, The Netherlands; 2 Bioinformatics and Statistics group, Department of Molecular Biology, Netherlands Cancer Institute, Plesmanlaan 121, 1066 CX Amsterdam, The Netherlands

## Abstract

**Background:**

Tumors have been hypothesized to be the result of a mixture of oncogenic events, some of which will be reflected in the gene expression of the tumor. Based on this hypothesis a variety of data-driven methods have been employed to decompose tumor expression profiles into component profiles, hypothetically linked to these events. Interpretation of the resulting data-driven components is often done by post-hoc comparison to, for instance, functional groupings of genes into gene sets. None of the data-driven methods allow the incorporation of that type of knowledge directly into the decomposition.

**Results:**

We present a linear model which uses knowledge driven, pre-defined components to perform the decomposition. We solve this decomposition model in a constrained linear least squares fashion. From a variety of options, a lasso-based solution to the model performs best in linking single gene perturbation data to mouse data. Moreover, we show the decomposition of expression profiles from human breast cancer samples into single gene perturbation profiles and gene sets that are linked to the hallmarks of cancer. For these breast cancer samples we were able to discern several links between clinical parameters, and the decomposition weights, providing new insights into the biology of these tumors. Lastly, we show that the order in which the Lasso regularization shrinks the weights, unveils consensus patterns within clinical subgroups of the breast cancer samples.

**Conclusion:**

The proposed lasso-based constrained least squares decomposition provides a stable and relevant relation between samples and knowledge-based components, and is thus a viable alternative to data-driven methods. In addition, the consensus order of component importance within clinical subgroups provides a better molecular characterization of the subtypes.

## Background

Gene expression data from tumors reflects many important clinical characteristics. For example, methodologies have been developed that can differentiate subtypes [1,2], predict disease outcome [3], and predict response to therapy [4]. Most of these aspects will also have a genetic basis, which is often unknown, and is typically not unveiled by purely data-driven techniques. Knowing the underlying molecular mechanisms is important if targeted therapies still need to be developed, and to determine whether a particular therapy is likely to be effective [5].

Based on the idea that tumors must be the result of an underlying mixture of oncogenic events [6,7], several attempts have been undertaken to decompose the gene expression profiles of tumors into components representing these oncogenic events. The components identified in these decompositions might then provide further leads towards understanding tumorigenesis. For example, Teschendorff et al. [8] have used Independent Component Analysis (ICA), and Principal Component Analysis (PCA), to decompose gene expression data from breast cancer samples. These methods are purely data-driven, and thus have the disadvantage that they do not employ any prior knowledge. For this type of decomposition, the relation between the components is pre-defined, e.g. they are required to be orthogonal/independent. In order to apply these methods a collection of tumor expression profiles is required. The choice of the number of components is typically based on the cumulative amount of variance explained by a set of components, which is largely arbitrary.

On a similar note, Brunet *et al*. [9] have used Non-negative Matrix Factorization (NMF) to decompose different leukemia subtypes. Similar to ICA/PCA, this method is data-driven, which makes the interpretation afterwards complicated. The main difference is that it places constraints on the decomposition: both the components vectors and weights are required to be non-negative.

Interpretation of the components/weights that are obtained using data-driven decomposition strategies is still very difficult. For example, the results can be compared to existing functional databases in order to attach an interpretation to the obtained components [8].

We would like to use the knowledge about relevant components directly in the decomposition. More specifically, we would like to use this knowledge by pre-defining the components used in the decomposition, rather than performing a post-hoc analysis of a fully data-driven result. Using this type of framework, i.e. employing components with a clear biological meaning, might result in a more meaningful decomposition and ease the interpretation afterwards.

Bild *et al*. [10], Acharya *et al*. [11], and Anders *et al*. [12] have used information about genetic perturbations to construct classifiers for perturbed vs wild type status. For every perturbation that was tested, they created a separate classifier, thus, they did not model possible interactions between these perturbations. Here, we use the expression profiles of a set of perturbed cell lines, and assume a linear model for their interaction, i.e. we model combinations of perturbations as a linear combination of the expression profiles. Thereby, we can include this type of knowledge directly into the decomposition. As opposed to post-hoc analyses of fully data-driven results (e.g. Teschendorff *et al*. [8]), several approaches have been developed to include information about pathways (e.g. GO [13] or KEGG [14]) as prior information into the analysis. For instance, Segal *et al*. [15] derived activity scores for gene sets by employing the hypergeometric distribution. Such an approach requires discretization of the expression data, which we preferably avoid since this might lead to a loss of information. Similarly, Chuang *et al*. [16] derived activity scores for subnetworks of a protein-protein interaction network by summing the Z-scores of the genes in such a subnetwork. A related approach is Gene Set Enrichment Analysis (GSEA), which was developed by Mootha *et al*. [17] and Subramanian *et al*. [18]. Later on, this approach was adjusted to allow the computation of the activity of a gene set in a single sample (Lamb *et al*. [19]). A common denominator among these approaches is that they treat each gene set separately, in the sense that the activity of a gene set in a particular sample is solved independently of the other gene sets. In contrast, we represent the expression of a given sample as the linear combination of the gene memberships of a predefined collection of gene sets.

We present a mathematical model (Constrained Least Squares Decomposition) that allows us to include knowledge driven components in the decomposition. More specifically, we model the expression profile of a single tumor as a weighted linear combination of a set of components. For these components, we use two sources of knowledge. First, we use the expression data from cell lines in which cancer associated genes have been perturbed, and second, we use gene sets that are representative of the six hallmarks defined by Hanahan *et al*. [6]. In order to keep the weights produced by the decomposition in an interpretable range, the process needs to be regularized. We do this by adding constraints on the weights, and introduce a Lasso regularization parameter [20].

We use the proposed model and the expression profiles of cell lines in which cancer associated genes were activated to decompose the gene expression profiles of genetically manipulated mice. Since the mutation status of these mice is known, and the mutated genes correspond to the genes perturbed in the cell line experiments, a direct performance comparison can be made. Next, we decompose the expression profiles of a set of breast tumors taken from six independent datasets, for which no mutation status is known, but where a set of clinical parameters, including disease and survival endpoints are known. Moreover, when changing the regularization parameter, we show that consensus patterns emerge in the order in which the weights become non-negative. The results show that tumors can be stratified into several subgroups, each characterized by a unique perturbation profile, which are associated with distinct outcomes. This is a powerful approach since it allows the characterization of subtypes based on specific molecular aberrations, and allows a more directed search for targeted therapies.

## Methods

### Mathematical framework

In our decomposition, we assume that a linear combination of a set of pre-defined components (*C*_1 _to *C*_*x*_) describes the gene expression observed for, for instance, a human tumor sample **y**. This implies that the gene expression of a sample can be written as a weighted summation over a set of components.

Mathematically the model can be defined as:

(1) **y **= **Cw**

where **y **is a column vector representing the gene expression that needs to be decomposed, **C **represents a matrix of individual component vectors (each column a component, *C*_*i*_), and **w **is a column vector of weights. In the linear model the weights in **w **reflect the extent to which the sample **y**, resembles the expression of the components that are in **C**. We assume that the weights in **w **are the same for all genes (i.e. for all rows in **y**, and **C**).

For a given sample (**y**) and matrix of component vectors (**C**), we can obtain an estimate of **w **by minimizing the Mean Square Error (MSE). The MSE (*ϵ*_*MSE*_) is defined as:

(2) $$\epsilon_{MSE}=\arg\underset{w}{\min}||(y-Cw)|{|}^{2}.$$

Without any constraints, a solution to Equation 2 can be found using the Moore-Penrose generalized Pseudoinverse ([21]), defined as:

(3) **w **= (**C**^*T *^**C**)^-1 ^**C**^*T *^**y**

(4) **w **= **C**^† ^**y**

where ^*T *^indicates a matrix transposition, ^-1 ^indicates a matrix inversion, and ^† ^indicates the pseudoinverse of a matrix. In the remainder of this paper, we refer to this variant as '*U*' (Unconstrained).

Without any constraints the weights in **w **are unbounded. As a result, weights in **w **might not have any biological relevance. For instance, it is hard to interpret negative weights in **w**, implying that the expression profile of a given component has a negative contribution to the reconstructed sample. Thus, a logical step is to include a variant, that ensures non-negativity of the weights in **w**, similar to an NMF approach [9]. This changes Equation 2 to

(5) $$\begin{array}{c} \epsilon_{MSE}=\arg\underset{w}{\min}||(y-Cw)|{|}^{2}\\ \text{subject to}\\ w\geq 0. \end{array}$$

In the remainder of this paper, we refer to this variant as '*P*' (constrained with positive weights). Apart from constraints on **w **we also consider a regularization term. For instance the L1-norm is an often applied form of regularization (Lasso, [20]). This regularization shrinks weights such that they become exactly zero, allowing the conclusion that the associated component vectors in **C **do not contribute to the reconstruction at all. This results in the remaining components with non-zero weights being 'selected'. In the spirit of the L1-norm, we introduce a constraint based variant that restricts the L1-norm to 1. That is, we include a variant for which the weights in **w **sum to 1. This changes Equation 2 to

(6) $$\begin{array}{c} \epsilon_{MSE}=\arg\underset{w}{\min}||(y-Cw)|{|}^{2}\\ \text{subject to}\\ \sum w=1\\ w\geq 0. \end{array}$$

We will refer to this variant as '*S*' (constrained with positive weights that sum to 1).

**Figure 1:**
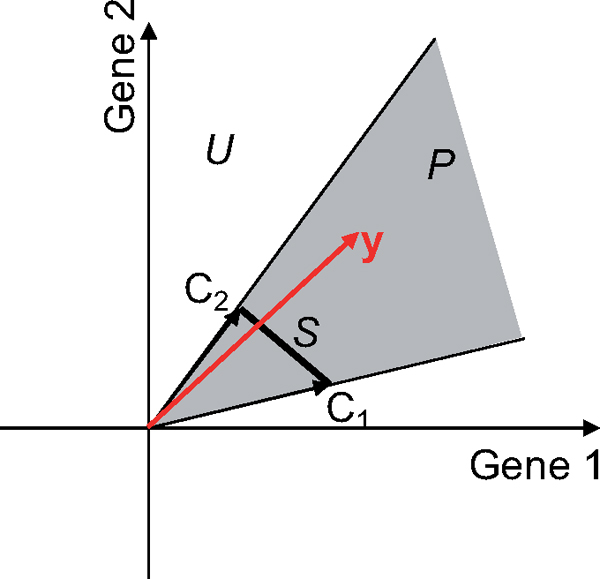
**Example of the solution space**. Ranges of solutions that can be produced for each of the three variants of constraints, for an example with two genes. The red arrow represents a gene expression profile to be approximated (**y**). The black arrows indicate two components *C*_1 _and *C*_2 _(i.e. columns in **C**). The unconstrained variant (*U*) can reconstruct any point in this Gene1- Gene2 space (determined system). The *P *variant can only reconstruct points in the grey area, which corresponds to non-negativity of the weights for the two components. Similarly, the *S *variant can only reconstruct points on the line joining the two components.

These different variants lead to different regions of possible solutions in the gene expression space, as indicated in Figure 1.

In addition, we also considered the option where we include a Lasso term into the variant with positive weights. This way, equation 2 changes to:

(7) $$\begin{array}{c} \epsilon_{MSE}=\arg\underset{w}{\min}||(y-Cw)|{|}^{2}+\lambda \sum \left|w\right|\\ \text{subject to}\\ w\geq 0 \end{array}$$

We will refer to this method as '*L*' (constrained with positive weights and Lasso term). Given the non-negativity constraint, the solution to Equation 7 is fairly simple. We appended a row to the matrix **C **with all elements set to *λ*, and append the target vector **y **with a zero.

The setting of the *λ *parameter will influence the weights that are obtained in **w**. Setting *λ *to infinite will result in an all zero **w **vector. Progressively lowering *λ *will result in an ordering in which the individual weights become non-zero [22]. Eventually, when *λ *is set to 0, the solution will be equivalent to the '*P*' solution, with up to all components having a non-zero weight.

**Figure 2:**
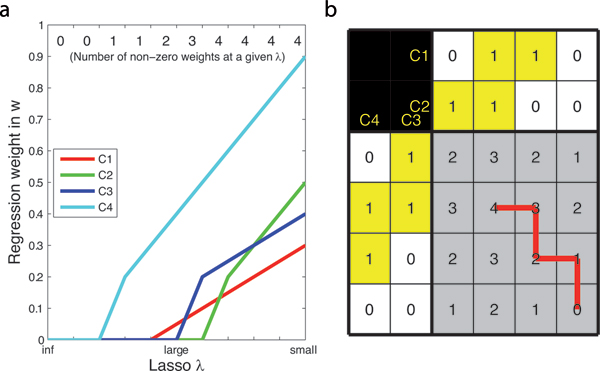
**Visualization of the Lasso shrinkage**. Example showing how the shrinkage of weights by the Lasso regularization is visualized. Let's assume we have a hypothetical case with four components, labeled C1 to C4. In subplot a on the left, we show an example of the weights in **w **as a function of *λ *(in analogy to [20]). In the top row of plot a we indicate the total number of non-zero weights. Then, subplot b on the right shows the table that is used to depict the order in which the weights, **w**, turn non-zero under Lasso regularization. The two rows at the top and the two columns to the left indicate whether a particular weight is non-zero (1, yellow cell shading), or zero (0, white cell shading). Numbers in the table (gray shaded area) indicate the combined number of non-zero weights in **w**, that is, all 16 (i.e. 2^4^) states are shown (possible combinations with 0 up to 4 weights being non-zero). There are 24 (i.e. 4!) possible unique paths to go from 0 to 4 non-zero weights. These paths can be traced in the plot assuming the left/right edges and top/bottom edges of the table are connected. We start with *λ *= inf, and slowly decrease *λ*. For an infinite *λ *the resulting **w **vector will be all-zero (bottom right in the table shown in subplot b). At a slightly lower *λ *one of the four weights will be the first to become non-zero. By lowering *λ *to zero, up to four weights will be non-zero. In subplot a on the left, the weights turn non-zero in the following order: C4, C1, C3 and lastly C2. The corresponding trajectory is depicted in subplot b on the right using the red line.

We hypothesized that there is a relation between the order in which the weights become non-zero, and the biology of the sample. That is, the first weight that becomes non-zero will be the most important, and each additional weight that becomes non-zero is less and less important. We hypothesize that the order of importance might be different for different clinical subgroups of tumors. We visualized the order in which the weights become non-zero by means of an adjusted Karnaugh map [23], See Figure 2 for a detailed example.

For each of the variants a constrained least squares optimization problem needs to be solved. To this end, we employed the Mosek optimization toolbox for Matlab [24]. This toolbox allows any number of equality and inequality constraints to be set, and employs an interior point algorithm.

### Datasets

#### HMEC dataset

We used a previously published dataset (Bild *et al*, [5]) which contains gene expression measurements of 45 Human Mammary Epithelial Cell cultures (HMECs) samples. These HMEC samples have been perturbed by an adenovirus, resulting in five different perturbations in genes (upregulation), namely in Myc (n = 10), Ras (n = 10), E2F3 (n = 9), Src (n = 7), and BCatenin (n = 9). These samples were analyzed on an Affymetrix Human Genome U133 Plus 2.0 Array, containing 54613 probes.

#### Mouse dataset

We used a previously published dataset (Bild *et al*, [5]) which contains 28 mouse samples. These mouse samples belong to five classes with different perturbations, namely in Myc (n = 5), Ras (n = 3), Rbnull (n = 6), Her2 (n = 7), and a Wild type (n = 7) class which serves as reference. These samples were measured on an Affymetrix array, containing 13179 probes.

#### MCF7 dataset

Creighton *et al*. [25] created a gene expression dataset from MCF7 (breast cancer) cell line samples. These cell lines were transfected with constitutively active RAF, MEK, ERBB2, and EGFR (overexpression). Each transfection was measured in triplicate, resulting in 12 arrays. Measurements were performed using the Affymetrix Human Genome U133A array, containing 22215 probes.

#### Human dataset

We used a collection of six publicly available breast cancer datasets (Reyal *et al*., submitted). These six datasets were all measured on the Affymetrix Human Genome U133A Array, containing 22215 probes. In total this combined dataset contains 509 samples for which distant metastasis free survival data (DMFS), ER status, and Hu *et al*. [2] subtype information is available.

#### Matching probes across the datasets

Given our four datasets, we want to decompose the mouse samples using the HMEC data as components, and, similarly, the human data using the MCF7 data as components. The samples from these four datasets originate from different organisms, and were measured on different platforms. In order to facilitate the decomposition, we have to match the probes from the mouse and HMEC data. To do this, we used the Chip Comparer utility [26], from Bild *et al *[5]. This way, we mapped these two datasets to a common set of 4383 genes. In case multiple probes mapped to one of the common genes, we selected the probe with the largest variance. Since the data were measured on different platforms, it is required to normalize each dataset separately. We applied mean-variance normalization per gene per dataset.

Both the human data and the MCF7 data was measured using the Affymetrix Human Genome U133A Array, eliminating the need to apply any probe matching. To normalize them, both datasets were median centered per gene prior to the analysis.

#### Gene sets

A collection of gene sets were gathered from the respective repository websites of GO [13], KEGG [14], and Reactome [27]. In total we gathered 7718 gene sets (GO: 6788, KEGG: 202, Reactome: 728, downloaded April 17, 2008). Based on their description, we assigned gene sets to the Hanahan hallmarks. For four hallmarks (Apoptosis, Angiogenesis, Growth, and Replication) we found associated gene sets. In our analysis, we used 5 gene sets that were associated with Apoptosis, 5 for Growth, 3 for Angiogenesis, and 3 for DNA replication. In Additional File 1, we provide a list of the gene sets and their Hanahan hallmark.

## Results and Discussion

### Decomposing mouse data into HMEC components

First, we decomposed the 14 mouse tumors into the available five classes of HMEC samples. To do this, we construct a **C **matrix, where each column consists of the class-means of the five perturbation classes represented in the HMEC samples (Myc, Ras, E2F3, Src, and BCatenin). It is unlikely that a perturbation will have an effect on all genes, causing many genes to be irrelevant with respect to a specific perturbation, consequently only contributing noise to the modeling problem. Therefore, we also applied a feature selection step on the HMEC data. We were most interested in genes that distinguish one of the HMEC classes from the other four. Therefore we ranked, for each of the classes, the genes based on their ability to discriminate between that class and the remaining classes. We employed the absolute signal to noise ratio (SNR) as ranking criterion. Next, we selected the top *n *genes for each of the five ranked lists, and then took the union of these five lists. Of course, alternative feature selection procedures can be employed, but they are beyond the scope of the current analysis. Using the set of genes selected in the feature selection step, and each of the mouse samples as target in **y**, we applied each of the four decomposition variants, *U*, *P*, *S*, and *L *(see methods section). After applying the models, we assign each mouse sample to the HMEC class which has the highest absolute *w*_*i*_. Since we know the mutation status of the mouse samples, we can compare the class assignment from the different variants to the known mutation status. To evaluate the predictive accuracy we only employed the three classes in the mouse dataset for which an equivalent class is present in the HMEC data. Therefore, we used the classes Myc, Ras and E2F3 (which is equivalent to Rb-null in mice), from the mouse data.

**Figure 3:**
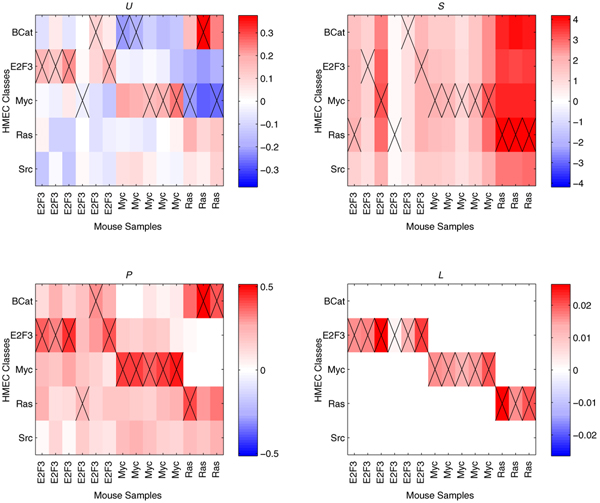
**Results on HMEC-Mouse data**. Heatmaps showing the **w **vectors for the decomposition of the mouse samples into the HMEC classes. The four heatmaps correspond to the four decomposition variants, *U*, *P*, *S*, and *L*, as indicated above each of the heatmaps. Each heatmap lists the 14 mouse samples along the x-axis, and the five HMEC classes along the y-axis. The heatmap reflects the **w **vectors that were obtained by decomposing each mouse sample separately. Each column contains one cross, indicating the largest absolute weight in that particular **w **vector, and thus the class assignment for that particular mouse sample. These solutions correspond to the case where the union of top 70 most differentiating genes for the separate one-versus-rest HMEC class comparisons are employed to represent the HMEC classes.

Figure 3 shows heatmaps indicating the obtained **w **vectors for all 14 mouse samples, as obtained by each of the four decomposition variants.

The **w **vectors found using the *U *variant have both positive and negative weights. Several mouse samples are incorrectly classified, most notably the Ras samples, which are all incorrect (i.e. their largest *w*_*i*_, indicated by crosses in Figure 3, does not correspond to the RAS HMEC class). Next, in the *P *variant, where all weights are constrained to be positive, all **w **vectors are almost identical for all mouse samples. This is a clear disadvantage, since no clear distinction between the mouse samples is made. For this variant, classification of the E2F3 samples turns out to be most difficult. Constraining all weights to sum to one (variant *S*), results in a distinctly different set of **w **vectors, but in terms of performance it is equal to variant *P*.

The lasso-based variant (*L*) provides the most desirable output, positive weights for the correct classes, and zeros everywhere else. Of course the result depends on the setting of *λ*, which was chosen such that a single non-zero weight is left for each of the mouse samples. This single remaining non-zero weight is what we hypothesize to be the most important weight.

**Figure 4:**
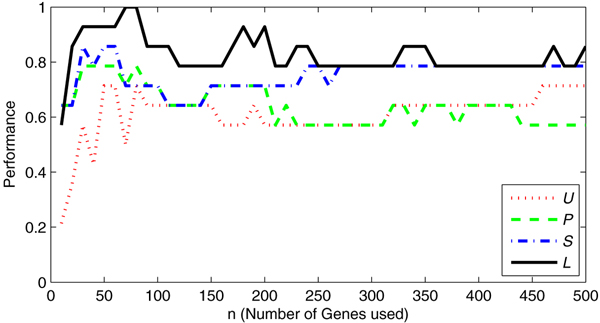
**Performance on the HMEC-Mouse data**. Performance (y-axis) of the four decomposition variants (*U*, *P*, *S*, and *L*), for a range of the selected number of genes *n *(along the x-axis). The performance indicates the fraction of mouse samples for which the largest absolute weight in **w **corresponds to the correct HMEC class. The number of genes, *n*, was varied from 10 to 500 in steps of 10.

Of course, the set of genes used in the decomposition influences the results. To investigate this, we inspected the performance of the four decomposition variants relative to the number of genes *n *that is selected in each one-versus-the-rest rankings. We define performance as the fraction of mouse correctly classified mouse samples, i.e. assigned to the HMEC class with the correct corresponding perturbation. We varied the number *n *from 10 to 500 genes. Figure 4 shows the resulting performance curves for each method. It is clear that the lasso-based method outperforms the other methods over the entire range of *n*, and reaches the best performance around 70 to 80 genes.

### Decomposing human data into MCF7 components

For the collection of 509 human breast cancer samples, we applied a decomposition into four MCF7 classes. Thus, we used the human samples as **y **vectors, and created a **C **matrix where the mean vectors of the four MCF7 formed the columns. We used the *L *variant to decompose the samples, since that showed the best performing decomposition on the mouse-HMEC data. We applied a feature selection step similar to that employed for the mouse-HMEC data to select the genes that are most discriminating between the four MCF7 classes. We employed the top 70 genes and set *λ *to 15% of the total number of genes, since these settings resulted in the best performance in the mouse-HMEC decomposition.

Unfortunately, for the human breast cancer data there is no information with regard to presence/absence of mutations. Nevertheless, a multitude of other clinical parameters is available for most of these samples. For all samples the estrogen receptor (ER) status, distant metastasis free survival time, and Hu *et al*. [2] subtype information is available. Any link between these clinical parameters and the MCF7 components is interesting.

**Figure 5:**
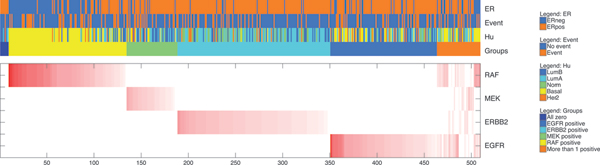
**Results on the MCF7-Human breast cancer data**. Output from the lasso-based decomposition variant (*L*), on 509 human breast cancer samples. The red and white heatmap indicates the **w **vectors for all samples (*n *= 70). Samples with the same single non-zero weight are grouped together. For the small group of samples on the left, all weights are zero, whereas for the samples on the right more than one element of **w **is non-zero. The ER status, whether a metastasis event occurred, and the subtyping according to Hu *et al*. are indicated by the top three colored rows above the heatmap. The groups formed based on which weights are non-zero are indicated in the fourth row (i.e. derived from this heatmap).

**Figure 6:**
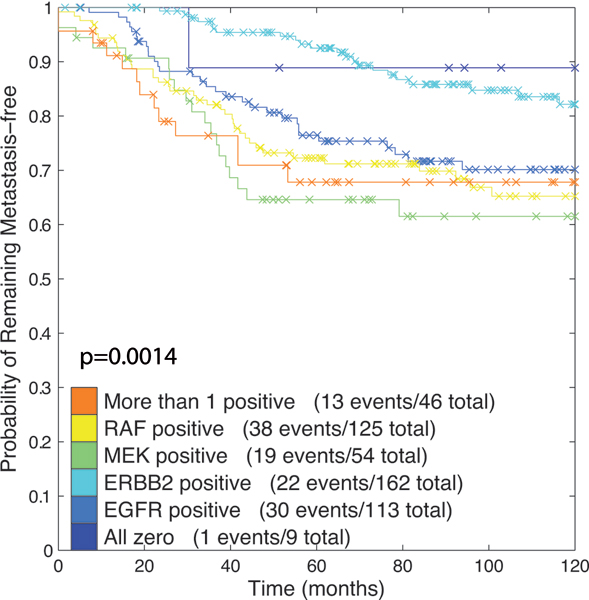
**Kaplan-Meier plot on the Human breast cancer data**. Kaplan-Meier plot indicating the difference in disease free survival characteristics of the six groups discerned on the 509 human samples. The samples were grouped into six groups based on the number of non-zero weights. Group 1 consists of a small set of samples which were assigned all-zero weight vectors. Groups 2 to 5 consist of samples with a single non-zero weight (the group being determined by the weight being non-zero). Group 6 consists of the samples with more than one non-zero weight. The p-value corresponds to the logrank test.

Figure 5 shows a heatmap of the **w **vectors that are obtained by decomposing the human samples into the MCF7 samples. The samples were grouped into six groups based on the number of non-zero weights. Group 1 consists of a small set of samples which were assigned all-zero weight vectors. Groups 2 to 5 consist of samples with a single non-zero weight (the group being determined by the weight being non-zero). Group 6 consists of about 70 samples with more than one non-zero weight. From a clinical point of view, the outcome parameter is the most important. Therefore, we wanted to test whether there is a relation between these six groups of samples, as represented in Figure 5, and disease free survival. To do so, we created Kaplan-Meier curves for each of the six groups, see Figure 6. The difference in survival characteristics between these six groups is significant (*p *= 0.0014, logrank test). Thus, the *L *decomposition has provided clinically interesting groups of samples with distinct outcome characteristics.

**Figure 7:**
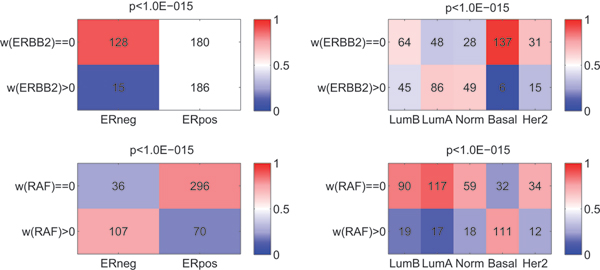
**Crosstables on the Human breast cancer data**. Four crosstables showing the relation between zero vs non-zero weights in **w **(rows), and a clinical parameter (columns). Each cell indicates the number of samples that fall into that category. Cell shading indicates the column-wise fraction. The p-values that are listed above each table correspond to a Chi-squared test for association between the variables indicated along the dimensions of the table.

Based on the grouping obtained in Figure 5, some relations with the clinical parameters are already visible. We employed the Chi-squared test to formally test the associations between each clinical parameter, and MCF7 class, see Figure 7. This allows us to test whether an association exists between the non-zero/zero weights and a given clinical parameter such as ER status. Figure 7 shows the most significant associations that were detected.

As shown in Figure 7, the majority of ER negative samples have a zero ERBB2 weight. At the same time, the ER positive samples are equally distributed between the ERBB2 present (non-zero weight) and ERBB2 absent (zero weight) groups. This association is highly significant (*p *< 10^-15^). Similarly, the majority of the samples from the Basal group, have a zero ERBB2 weight (*p *< 10^-15^). This confirms a previous observation that the Basal samples are predominantly triple negative, i.e. ERBB2, ER, and PR negative, Kreike *et al*. [28]. In addition, we made a Kaplan-Meier plot for the two groups obtained by splitting on ERBB2 weight status in (zero/non-zero). The difference in survival is clearly significant (*p *= 1.4*e *– 5, figure not shown).

Figure 7 also shows that the majority of ER negative samples have a positive RAF weight, and at the same time that most of the ER positive samples have a zero RAF weight. The association between RAF and ER status is highly significant (*p *< 10^-15^). Both the RAF and ESR1 (ER) genes are key players in the MAPK signalling cascade (result not shown, String database, string.embl.de). Thus our analysis confirms the close relation between these two genes.

The subtypes provided by Hu *et al*. [2] include a Her2 group. This group is of particular interest since this is equivalent to the ERBB2 class in the MCF7 dataset. It turns out that there is little to no correlation between these two assignments, only 15 out of the 46 Her2 samples have a non-negative ERBB2 weight (see Figure 7). Strikingly, the majority of samples with a non-negative ERBB2 weight are the Luminal A and normal-like samples. Another method has been published that allows the determination of the Her2 status solely based on 1 probe that shows a bi-modal expression distribution (Gong *et al*. [29]). We also determined the Her2 status using this method (results not shown). It turns out that there is limited to no correlation among the Her2 assignments, as derived by Hu *et al*. [2], Gong *et al*. [29], and our method. A potential explanation for this might be that the Hu *et al*. and Gong *et al*. Her2 subtype is defined largely by the Her2 expression itself, and much less by its downstream effects. Only a known ground truth can give an indication which of the three assignments best reflects the actual perturbation status of Her2. However, such data is currently not yet available.

### Order of Lasso shrinkage

**Figure 8:**
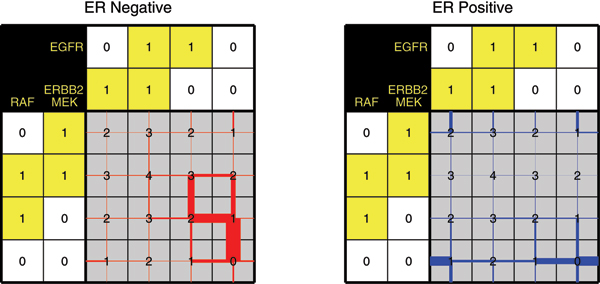
**Visualization of Lasso shrinkage in the ER subgroups**. Table indicating the order in which the weights in **w **become non-zero, when changing *λ*. The figure shows the results for the ER negative (left) and ER positive (right) groups separately. The linewidth is proportional to the total number of samples in the ER negative/positive subgroup, respectively.

Next, we inspected the effect of the regularization parameter on the order in which the weights in **w **become non-negative. Thus, we obtain tables with trajectories for each of the 509 samples, similar to the example shown in Figure 2. In order to create an aggregate plot of all trajectories across 509 samples, we created a slightly adapted representation, see Figure 8. More specifically, the linewidth of the red/blue lines in Figure 8 is proportional to the fraction of tables (samples) that have that link, relative to the total number of samples. For example, let's assume there are 300 out of 500 samples that traverse 0 to 1 based on a positive weight for RAF (i.e. upwards in Figure 2), then that line will be plotted with 0.6 times the maximum linewidth.

Figure 8 shows that there is a clear consensus in the ER negative table. For many ER negative samples, first the RAF weight becomes positive, followed by the MEK weight as second and EGFR weight as third (or alternatively EGFR as second, and MEK as third). For the ER positive samples, the trajectories are much more diverse, and no clear consensus is seen. This implies that the group of ER negative samples is more coherent in terms of the order in which these samples are decomposed into the separate components.

### Decomposing human data into gene sets

As an alternative source for knowledge driven components, we used gene sets. More specifically, we used gene sets that were linked to the hallmarks of cancer described by Hanahan *et al*. [6] (see Materials and Methods section). This was done by creating a **C **matrix with these 16 gene sets as components. Thus, the **C **matrix has 16 columns, one for each gene set. For each gene set (i.e. each column) the entries in **C **are set to 1 for gene that is part of that gene set, and set to zero otherwise. We used this **C **matrix with gene sets to decompose the 509 breast cancer samples (where the expression profile of the tumor samples were iteratively inserted in the **y **vector). Once again we applied feature selection, by performing the decomposition using only those genes that are assigned to at least one of the gene sets used.

**Figure 9:**
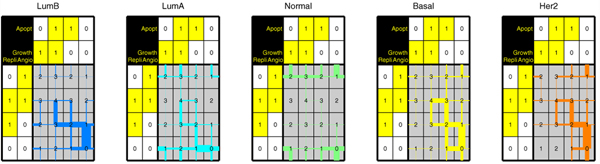
**Visualization of Lasso shrinkage in the Hu subtypes**. Table indicating the order in which the weights in **w **become non-zero, when changing *λ*. The figure shows the results for the human samples split over the five subgroups defined by Hu et al. [2]. Gene sets that correspond to these four Hanahan hallmarks were chosen as components. The linewidth is proportional to the total number of samples in that clinical subgroup. Apopt: Apoptosis; Growth: Growth; Angio: Angiogenesis; Repli: DNA Replication.

Figure 9 shows the resulting trajectories associated with each of the groups defined by Hu *et al*. A hallmark is now considered to be zero when the weights of all components linked to that hallmark are zero. In most subgroups a consensus trajectory can be discerned. The consensus order of importance in the Her2, LuminalB, and Basal subtypes is similar: first replication, second apoptosis, followed by angiogenesis and growth. In the LuminalA group the order the first two are flipped, that is, first apoptosis, and second replication attains a non-zero weight. In all four of these subgroups, a reasonable part of the samples ends up with a vector having four non-zero weights.

On the other hand, in the normal-like group, there is no clear consensus, and samples first get a non-zero weight for either one of apoptosis, growth, angiogenesis. Only very few samples obtain a non-zero weight for replication. Consequently, almost none of the normal-like samples get to the stage with four non-zero weights. This signifies a discriminating characteristic of the normal-like samples with respect to the other four categories. This is slightly contradictory to the original Hanahan *et al*. [6] hypothesis, which states that a tumor must have obtained all six of the hallmarks. However, the normal-like group of breast cancer samples, is also the one with the best survival characteristics [2]. Perhaps this better survival is, in part, explained by the fact that the replication hallmark is not as active as in some of the other breast cancer subtypes.

## Conclusion

We described a linear model which links a set of knowledge-derived expression vectors to the expression profile of samples, potentially with unknown mutation status. When benchmarked on data from HMECs and Mouse, the lasso-based method outperforms the best. Moreover, the lasso-based method is relatively insensitive to the setting of the regularization parameter *λ*, and performs well for the entire range of genes (*n*) that is selected. Thus, the proposed lasso-based constrained least squares decomposition provides a parameter-insensitive and accurate assignment of mutation status to samples.

On the collection of 509 human breast cancer samples, we found several associations between the molecular component class the samples were assigned to and the clinical parameters. This includes both new associations (RAF with ER status), and known associations (ERBB2 weight zero with ER negativity/Basal subtype). Thus, the proposed decomposition framework has a clear capability to unveil relevant relations between the molecular components and the human samples, for which no mutation status is known. Using gene sets as components has unveiled different consensus trajectories of appearance for the components representing the Hu *et al*. subtypes when changing the regularization parameter *λ*. We hypothesize that these trajectories provide insight into the key events that gave rise to the tumor and might shed light on the future behavior of the tumor, including how it will react to therapy.

A main advantage of our method is that it allows the incorporation of knowledge derived components, which is not possible for most data-driven methods. Moreover, it is possible to do the decomposition for even just one sample (i.e. a single **y **vector). This is not possible for, for example, PCA, where a group of **y **vectors is required. A limitation of our method is that it requires a set of components derived from knowledge. Of course, for interpretation of the data-driven components, this knowledge has to be available as well. Thus, the knowledge based decomposition presented here is a viable alternative.

## Competing interests

The authors declare that they have no competing interests.

## Authors' contributions

MHV, LFAW, and MJTR contributed to the conceptual design of the study. MHV performed the analysis. MHV, LFAW, and MJTR wrote the manuscript.

## Supplementary Material

Additional file 1 Names of the gene sets used. In this table, we indicate which gene sets were used for which of the Hanahan hallmarks. Click here for file
